# Lacking Control over the Trade-Off between Quality and Quantity in Visual Short-Term Memory

**DOI:** 10.1371/journal.pone.0041223

**Published:** 2012-08-08

**Authors:** Alexandra M. Murray, Anna C. Nobre, Duncan E. Astle, Mark G. Stokes

**Affiliations:** 1 Department of Experimental Psychology, University of Oxford, Oxford, United Kingdom; 2 Oxford Centre for Human Brain Activity, University of Oxford, Oxford, United Kingdom; 3 Medical Research Council Cognition and Brain Sciences Unit, Cambridge, United Kingdom; University of Groningen, Netherlands

## Abstract

Visual short-term memory (VSTM) is limited in the quantity and quality of items that can be retained over time. Importantly, these two mnemonic parameters interact: increasing the number of items in VSTM reduces the quality with which they are represented. Here, we ask whether this trade-off is under top-down control. Specifically, we test whether participants can strategically optimise the trade-off between quality and quantity for VSTM according to task demands. We manipulated strategic trade-off by varying expectations about the number of to-be-remembered items (Experiments 1–2) or the precision required for the memory-based judgement (Experiment 3). In a final experiment, we manipulated both variables in a complementary way to maximise the motivation to strategically control the balance between number and the quality of items encoded into VSTM. In different blocks, performance would benefit most either by encoding a large number of items with low precision or by encoding a small number of items with high precision (Experiment 4). In all experiments, we compared VSTM performance on trials matched for mnemonic demand, but within contexts emphasising the quality or quantity of VSTM representations. Across all four experiments, we found no evidence to suggest that participants use this contextual information to bias the balance between the number and precision of items in VSTM. Rather, our data suggest that the trade-off may be determined primarily by stimulus-driven factors at encoding.

## Introduction

Capacity limits in visual short-term memory (VSTM) can be characterised by two key parameters: the number of memory items and the quality with which they are remembered. Within the capacity limits of VSTM there is a trade-off between quality and quantity. Increasing the number of memory items reduces the quality of individual memory representations [1], [2]. Although this trade-off is well established, it remains unclear whether the relative allocation of VSTM capacity is under top-down control.

The relative trade-off between quality and quantity in VSTM has sparked a debate regarding the flexibility over the allocation of VSTM resources [3]. On the one hand, fixed-resolution accounts suggest that VSTM resources are discretely allocated to fixed item “slots” with high precision. When all slots are filled, no information about the additional items is maintained, and there is little or no flexibility in the trade-off between quantity and quality of representations [2], [4]–[6]. A related account further proposes that if the number of to-be-represented items is below the maximum number of slots, then items can be represented by multiple slots. This “slots + averaging” model would predict that precision can increase when only a few items are represented. Alternatively, flexible resource models e.g. [7] do not impose an upper item limit, but suggest that VSTM resources can be allocated flexibly to accommodate increasing numbers of behaviourally relevant items, albeit with increasingly less precision [1], [8].

We suggest that both limits could be optimised for behaviour [9]. In some cases it may be important to represent more than one item in VSTM, even at the cost of precision, therefore VSTM resources should be shared amongst more than one task-relevant item VSTM. However, it is also important that each item is represented with sufficient quality to guide behaviour – a very large number of very imprecise representations would serve no obvious behavioural advantage. According to this constraint, the maximum number of items should be limited to ensure a minimum level of representational quality [10]. However, it remains unclear whether this trade-off is fixed, or can it vary flexibly with changing task demands?

An extensive literature demonstrates that prior knowledge of up-coming behavioural demands can be used to optimise processing accordingly. Top-down signals, particularly from prefrontal cortex [11], can bias the type of information that is processed within the visual system. Preparatory attention directed to spatial locations or attributes can facilitate task-relevant sensory processing by up-regulating specific cortical areas that are predicted to be task relevant [12]–[15]. Similarly, in the VSTM literature, behavioural and neuroimaging experiments have revealed the beneficial effects of orienting attention to the location or features of task-relevant items during encoding [16]–[19]. In Murray et al. [18], for example, spatially predictive cues triggered anticipatory brain states that were associated with selecting a sub-set of items for encoding into VSTM. There was no evidence that attention influenced the precision of VSTM encoding, see also [2]. However, previous experiments have not specifically manipulated attention to the task demands in a way that would motivate different settings of the trade-off between the number and the quality of items represented in VSTM. In these experiments, participants could not predict in advance the relative demands regarding the number or precision for VSTM, but only which items are more likely to be relevant to behaviour.

One exception is a recently published study by Zhang and Luck [20] who manipulated the degree of precision required to respond in a delayed colour-discrimination task. Participants either responded by clicking on a continuous colour wheel, requiring high precision, as per [2] or by selecting among a number of alternative colour wedges (requiring comparatively lower precision). Zhang and Luck [20] found that participants were unable to trade-off resolution and capacity in VSTM. The current study further explores this intriguing and potentially important null effect.

To determine whether the quantity-vs.-quality trade-off in VSTM is under flexible, strategic control, we conducted a series of experiments in which predictive information was provided to participants regarding either the likely number of items in an upcoming array (Experiments 1 and 2), the precision with which they needed to be recalled (Experiment 3), or a combination of the two (Experiment 4). The predictive cues indicated the likely demands in a precision-variant of a change-detection task in which the degree of change between memory item and probe is varied parametrically [1], [18]. Critically, change discrimination for small differences requires high precision (or high-quality) memory representations, whereas discrimination of large changes could be performed with coarser representations. Moreover, if an item is not represented at all, then performance should be at chance, irrespective of the change magnitude. By modelling behaviour as a function of change magnitude it is possible to estimate whether an item is represented in VSTM [2]; if it is, we can also estimate the precision of the mnemonic representation [1].

In this study, we ask whether it is possible to choose strategically whether to represent fewer items in order to encode each item with higher resolution or whether to represent more items by sacrificing the quality of each representation. Across the four experiments, however, we found a resounding lack of evidence to suggest that participants used the provided contextual information to influence the deployment of VSTM resources. Together, the results suggest that the allocation of VSTM resources is automatically determined by stimulus-driven factors, and in particular, the number of task-relevant items in the display. This negative result provides additional support for the null effect reported by Zhang and Luck [20], providing important convergent evidence that the trade-off between quality and quantity in VSTM is not biased by the foreknowledge of expected task demands.

## Experiment 1

The first experiment tested whether the trade-off between the quality and quantity of items in VSTM can be adjusted flexibly according to the task demands. Prior to the presentation of the memory array in each trial, participants were informed that the number of to-be-remembered items would be comparatively low (2 or 4) or high (4 or 6). If VSTM resource allocation is under flexible control e.g. [1], participants should be able to optimise the trade-off between quality and quantity to favour more precise representations when set size is expected to be small, and/or to encode more items by trading-off quality for quantity in anticipation of large set sizes. Accordingly, performance on trials with the common set size (4 items) should be more precise when participants anticipate a small set-size compared to a large set-size.

### Methods

#### Participants

Thirteen participants took part in Experiment 1; however, one was excluded due to low average behavioural performance (less than 55% accuracy). The remaining participants (3 female, 9 male; age range 19 and 32) were right handed as assessed by a handedness inventory [21] and had normal or corrected-to-normal vision. All participants in Experiment 1 were reimbursed £7 for participation in the experiment and provided written informed consent. The methods in this and in all subsequent experiments were approved by the Oxford Central University Research Ethics Committee (CUREC).

#### Task

**Figure 1:**
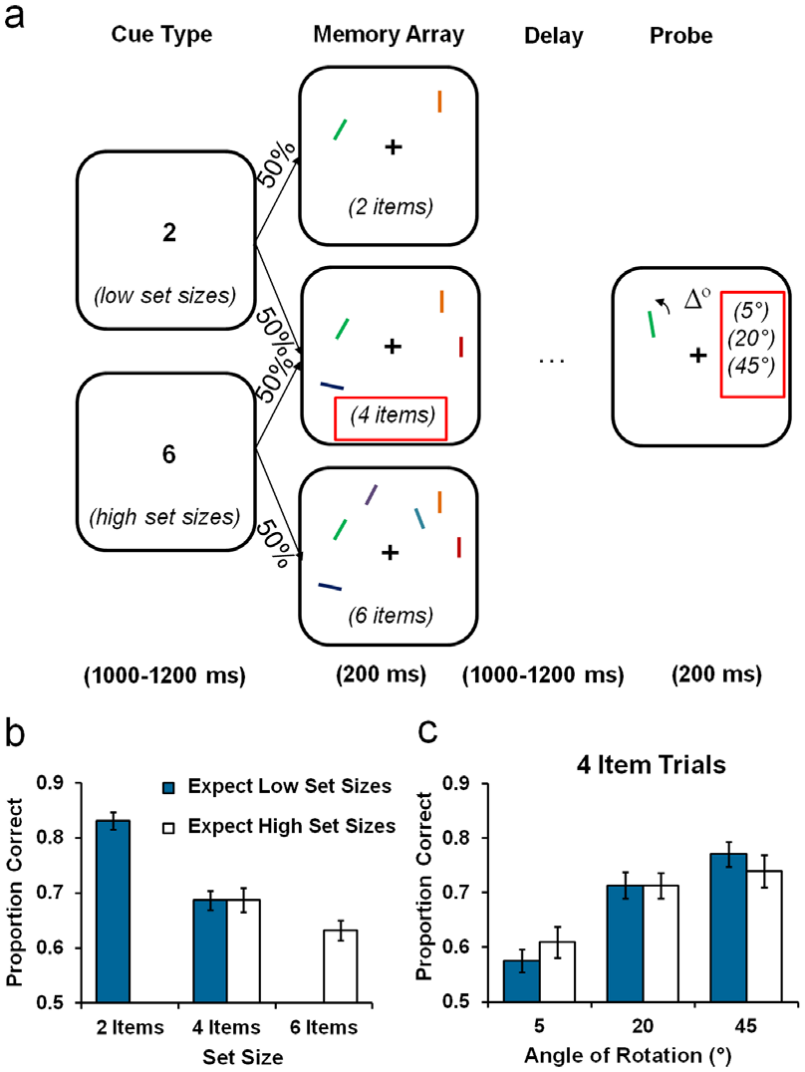
Task schematic and main results for Experiment 1. [a] A cue of either “2” or “6” was presented for 1000–1200 ms to indicate the likely set size of the upcoming array. The memory array was then presented for 200 ms and consisted of either 2 or 4 oriented lines in the *cue low set-size* condition, or 4 or 6 lines in the *cue high set-size* condition. After a variable delay (1000–1200 ms), a single item was re-presented for 200 ms, but rotated either 5°, 20° or 45° in a clockwise or anti-clockwise direction. Participants judged the direction of angular change. Red boxes indicate common comparison conditions between the Cue Type conditions at set size 4 and across all levels of rotation (5°, 20° and 45°). [b] The proportion of correct orientation change discriminations is shown as a function of the Cue Type condition and Set Size. [c] The proportion of correct orientation change discriminations for 4 item trials is shown as a function of the Cue Type condition and Angle of Rotation. Error bars represent ±1 *SEM*.

The experimental task (Figure 1a) was a variant of a VSTM precision task used by Murray, Nobre and Stokes [18], and based on the task introduced by Bays and Husain [1]. On each trial, participants were presented with a set-size cue for 1000-1200 ms, followed by a briefly presented array (200 ms) of randomly oriented coloured lines to encode. After a variable retention interval (1000-1200 ms), memory for a single item was tested at the end of the trial. The memory probe was identical to one of the lines from the memory array, but rotated about its central axis (angular change: ±5°, ±20° or ±45°). Participants judged whether the probe had rotated clockwise or anti-clockwise relative to the original memory item. Accuracy feedback was presented for 500 ms after response.

There were four trial types, defined by the range of set sizes predicted by the cue (low vs. high) and the number of items in the array. On *cue low set-size* trials, the memory arrays contained either 2 or 4 items (in equal numbers). On *cue high set-size* trials, arrays contained either 4 or 6 items (in equal numbers). All items within the memory arrays were equally distributed on both sides of the visual field. All trial types were randomised throughout the experiment. This design enabled us to compare performance on a common condition (4 items) to determine whether the anticipation of the upcoming set size modulated the strategic allocation of VSTM resources.

#### Stimuli

The task was programmed and presented using Presentation software [22]. All stimuli were presented against a black background. Before each trial, the cue was a “2” for *cue low set-size* trials or “6” for *cue high set-size* trials (each approximately 0.83° by 0.83°) in grey (RGB: 100, 100, 100), Arial 28pt font. The cue was present throughout the trial, until the offset of the probe. A grey fixation cross (0.32° by 0.32°) was presented in the centre of the screen between each trial. Colours were chosen at random (without replacement) from a set of eight highly discriminable colours (Red, Blue, Green, Cyan, Magenta, Yellow, Orange or White). Each line subtended approximately 1.43° of visual angle from base to tip, and a minimum distance of 2.49° between each stimulus ensured that there was no overlap, and reduced crowding effects. Participants pressed the right ctrl key for “clockwise” responses with their right hand and the left ctrl key with their left hand for “anti-clockwise” responses on a standard keyboard.

#### Procedure

Participants performed the computerised task in a quiet, dimly lit room viewing a CRT monitor (60 Hz refresh rate) at a distance of 90 cm. After completing 16 practice trials, participants performed 384 experimental trials. Participants were encouraged to use the cue to anticipate the likely number of items in the upcoming array.

#### Analysis

In experiment 1, data were first collapsed across clockwise and anti-clockwise rotations, leaving 32 trials per condition. The most relevant analysis compared recall accuracy at the common set size (4 items) according to Cue Type (cue high set-size vs. cue low set-size) and Angular Change (5°, 20°, 45°) using a repeated-measures analysis of variance (ANOVA). For completeness, two additional 2×3 repeated-measures ANOVAs also assessed performance for each Set Size (2 vs. 4, or 4 vs. 6 depending on the level of Cue Type) and Angular Change (5°, 20°, 45°) on the *cue low set-size* and *cue high set-size* trials separately (see Supporting Information S1). Greenhouse-Geisser corrected p-values are reported when sphericity could not be assumed (as assessed by Mauchly’s test). Post-hoc pairwise comparisons of means were used to interpret results of interactions when necessary, and were Bonferroni corrected for multiple comparisons.

### Results and Discussion

As illustrated in Figure 1b, performance varied as a function of Set Size, however, there was no apparent difference between Cue Type conditions at the common level of Set Size: 4 items. Statistical analysis of the performance on the common, 4-item condition, showed no significant main effect of Cue Type [*F*_1,11_ = 0.004, *p = *0.95] and no interaction between Cue Type and Angle of Rotation (*p = *0.14). However, there was a main effect of Angle of Rotation [*F*_1,11_ = 30.20, *p<*0.001], owing to lower performance for 5° compared to 20° (*p*<0.001) and 45° angles of rotation (*p<*0.001). Performance for 20° and 45° rotations was not different (*p* = 0.32).

If participants were able to use the cue information to optimise VSTM resource allocation, the precision of VSTM should vary as a function of the number of memory items that are likely to be presented [10]. However, the critical comparison at the common 4-item trials showed no such difference between the two types of cue. From these results, it would appear that mechanisms for top-down control did not, and/or were unable to, optimise the allocation of VSTM resources in anticipation of the array.

We considered two experimental aspects that could account for this null effect. Firstly, although the cue was on average predictive of a high or low set size, 50% of the time the cue was followed by the common condition (i.e., set-size 4). This could have reduced the efficacy of the cue. Secondly, control settings may not be flexible enough to accommodate the trial-by-trial changes in set-size probability. Trial-by-trial fluctuations may be too rapid for strategic shifts in VSTM resource allocation. To rule out these two potential explanations, Experiment 2 tested whether participants can optimise performance when quantity probability is blocked, rather than manipulated trial-by-trial. In addition, the proportion of trials with the common set size was reduced in order to increase the validity of the cued task expectations.

## Experiment 2

Experiment 2 tested whether the lack of effect in Experiment 1 could be attributed to an inability to modulate the required allocation of resources on a trial-by-trial basis. In this design, participants were instructed at the beginning of each block to expect either low or high set-size trials (*block low set-size* vs. *block high set-size*, respectively). In addition, the proportional weighting of the 2- and 6-item trials in the respective low and high set-size blocks was set to 66.7%. Finally, increasing the trial numbers also enabled psychometric modelling of the precision and probability of recall of representations across the two block types.

### Methods

#### Participants

Twenty volunteers (9 female, 11 male, aged between 20 and 34) were recruited for Experiment 2. All participants were right handed as assessed by a handedness inventory [21], had normal or corrected-to-normal vision, were remunerated £10 for their participation and provided written informed consent.

#### Task

**Figure 2:**
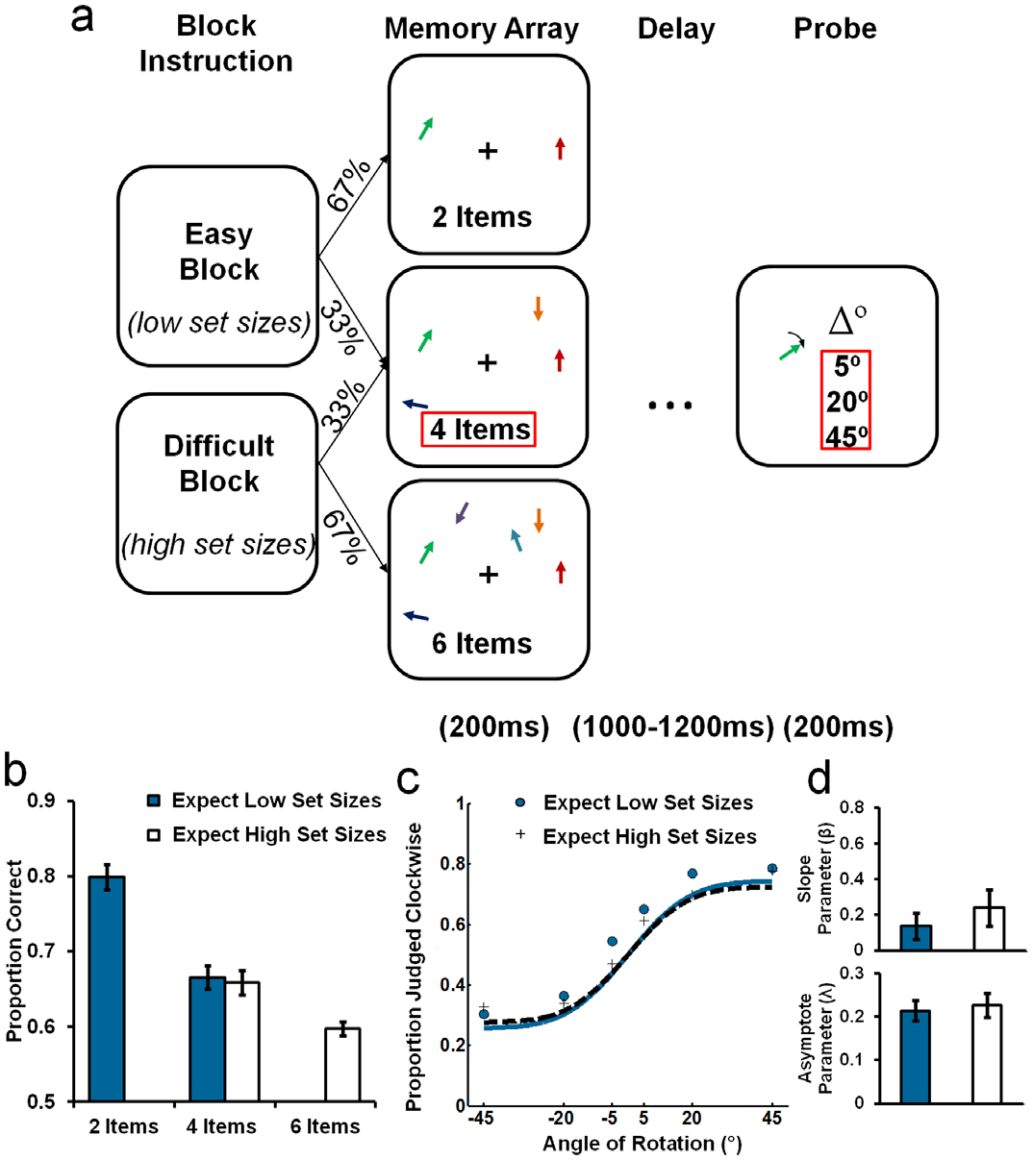
Task schematic and main results for Experiment 2. [a] In *low set-size* blocks participants attempted to encode 2 or 4 items (top) or 4 or 6 items in the *high set-size* blocks (bottom). Red boxes indicate common comparison conditions between the Block Type conditions: Angular Change (5°, 20° and 45°) and a set size of 4 items. [b] The proportion of correct orientation-change discriminations is shown as a function of the Block Type and Set Size. Data shown are the average across all trials. [c] Average proportion of clockwise responses as a function of Block Type and Angle of Rotation. To demonstrate, the lines represent the reconstructed model, fitted to the average data for the two Block Types. [d] Top: Mean of the individual slope parameter (β) estimates for both Block Type conditions. Bottom: Mean of the individual asymptote parameter (λ) estimates for both Block Type conditions. Error bars represent ±1 *SEM*.

The conditions of the task are illustrated in Figure 2a. The design, task, procedure and analysis were similar to those in Experiment 1, but with two important differences. Set-size probability was cued at the beginning of each block, and maintained for at least 108 trials, and the relative proportion of validly cued items was increased. On *block low set-size* trials (cued by an on-screen instruction: “Easy Block”), the memory arrays contained either 2 (66.67% of trials) or 4 items (33.33% of trials). *Block high set-size* trials (cued by an on-screen instruction: “Difficult Block”) contained either 4 (33.33% of trials) or 6 items (66.67% of trials) in the memory array. All items within the memory arrays were equally distributed on both sides of the visual field and presented for 200 ms. As in Experiment 1, this design enabled us to compare performance on a common condition (4 items) to determine whether expectation for relatively small or large numbers of items to encode modulates the strategic allocation of VSTM resources.

#### Stimuli

Memory arrays consisted of oriented coloured arrows as in [1], [18] rather than lines used in Experiment 1. Stimuli were presented against a light grey background (RGB: 192, 192, 192). Before each block, the text “Easy Block” (5.09° by 0.83°) or “Difficult Block” (6.37° by 0.83°) was presented in black, Arial 34pt font to cue participants to the likely number of items in each trial of the upcoming block. This instruction remained on screen until participants self-initiated the experimental block. A black fixation cross (0.32° by 0.32°) was presented throughout each trial, offsetting only when feedback was given. The colours of arrows were chosen at random (without replacement) from a set of eight highly discriminable colours (Red, Blue, Aqua, Magenta, Orange, Purple, Black or White). The spacing and size of the stimuli were approximately the same as Experiment 1. Responses were made by turning the lever of a custom-built response device clockwise or anti-clockwise, according to the direction of orientation change. Accuracy feedback was presented for 500ms after response.

#### Procedure

After completing 16 practice trials, participants completed 1080 trials, presented across 5 low and 5 high set-size interleaved blocks (order counterbalanced across participants). All other variables were randomised within blocks. Eye movements were monitored to ensure participants maintained fixation. Participants were instructed that the cue information presented at the beginning of each block provided information about the likely number of items per trial in the upcoming block.

#### Analysis

As in Experiment 1, the critical comparison was accuracy at the common level of Set Size (4 items) as a function of Block Type. In the first analysis, we submitted the data to a repeated-measures ANOVA, with factors for Block Type (*block low set-size* vs. *block high set-size*) and Angular Change (5°, 20°, 45°). In this experiment, we were also able to model behavioural data to estimate the two key mnemonic parameters: quality and quantity, indexed respectively by the slope and asymptote of the psychometric function [1], [2], [18]. Data were modelled for each participant using a cumulative normal distribution implemented by a custom Matlab toolbox [23]. Firstly, the observed distributions of responses “clockwise” were fitted to a cumulative Gaussian curve for each participant and condition. Responses were modelled as binary outcomes following:


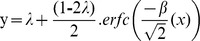


Where erfc is the complementary Gaussian error function, β is the slope of the curve, and λ is the asymptote [18]. The upper and lower bound recall performance reflects the maximum number of items in VSTM, assuming the comparison judgment is infinitely easy [2]. An estimate of the asymptote, therefore, provides an estimate of the maximum number of items available in VSTM at the time of the test probe. In contrast, the estimate of the slope provides an index of the precision of available memory representations [1]. Differences in slope and asymptote estimates were compared between the levels of Block Type using paired sample t-tests. For completeness, we also examined the effect of Set-Size and Angular within each level of Block Type (see Supporting Information S2).

### Results and Discussion

Memory performance in Experiment 2 is presented in Figure 2b. Analysis of performance on the common, 4-item condition revealed no significant main effect of Block Type [*F*_1,19_ = 0.49, *p = *0.50] and no interaction between Block Type and Angular Change [*F*_2,38_ = 1.45, *p = *0.25]. There was a significant main effect of Angular Change [*F*_1,19_ = 50.46, *p<*0.001] and follow-up analysis revealed that, for every comparison, accuracy was higher for larger angles of rotation (*p*s≤0.009).

Comparisons of the estimates of the modelling parameters also failed to reveal an effect of Block Type on performance at set-size 4. There was no significant difference between slope-parameter estimates [*t*_19_ = 1.45, *p* = 0.16; see Figure 2d] as a function of Block Type. If anything, this reflects a trend for higher precision when participants expect a large number of items. There was no significant difference in estimated asymptotes between the *block low set-size* and *block high set-size* trials [*t*_19_ = 0.32, *p* = 0.75; see Figure 2d].

Across two experiments, we found no evidence that foreknowledge of the likely number of memory items can be used to optimise the allocation of memory resources. This was the case when predictions were informed by trial-by-trial cueing or were blocked. Increasing the proportion of validly cued trials also did not influence the effect of quantity cueing. In Experiment 2, we were also able to estimate the psychometric parameters that reflect the precision and probability of recall. Again, there was no evidence to suggest that expectation of the upcoming size of the array could affect the allocation of resources.

These results provide accumulating evidence that foreknowledge of the likely number of memory items is not used to optimise the trade-off between VSTM quality and quantity at encoding. Rather, it would seem that observers allocate VSTM resources according to the actual contents of the memory array, irrespective of their expectations. In Experiment 3 we test whether foreknowledge of the likely *precision* required for successful recall can influence VSTM encoding. Because the actual precision is not known until the end of the trial, participants should use their predictions rather than the stimuli present to allocate resources and optimise encoding.

## Experiment 3

Experiment 3 followed the same structure as Experiment 2. However, instead of manipulating set-size probability, we manipulated the likely precision required to discriminate between the angular change: *low precision block* (45° or 20°) or *high precision block* (20° or 5°). As in Experiments 1 and 2, the key question was whether performance in the common condition (now angular change of 20°) varies as a function of blocked expectation. This design could be more effective in motivating optimal trade-off for VSTM encoding because, unlike set size, the required precision only becomes evident at the probe stage. If participants can optimise resource allocation during encoding, performance in the common condition (20°) should be more accurate in the high precision blocks relative to low precision blocks, owing to the higher expected demand for precise representations in those blocks.

### Methods

#### Participants

Twenty-three volunteers with normal or corrected-to-normal vision were recruited for Experiment 3 and provided written informed consent. Three participants were excluded due to poor behavioural performance (overall average accuracy less than 55%). The remaining participants (11 female, 9 male; aged between 19 and 34) were included in the analysis. All participants except one were right handed as assessed by a handedness inventory [21].

#### Task and stimuli

**Figure 3:**
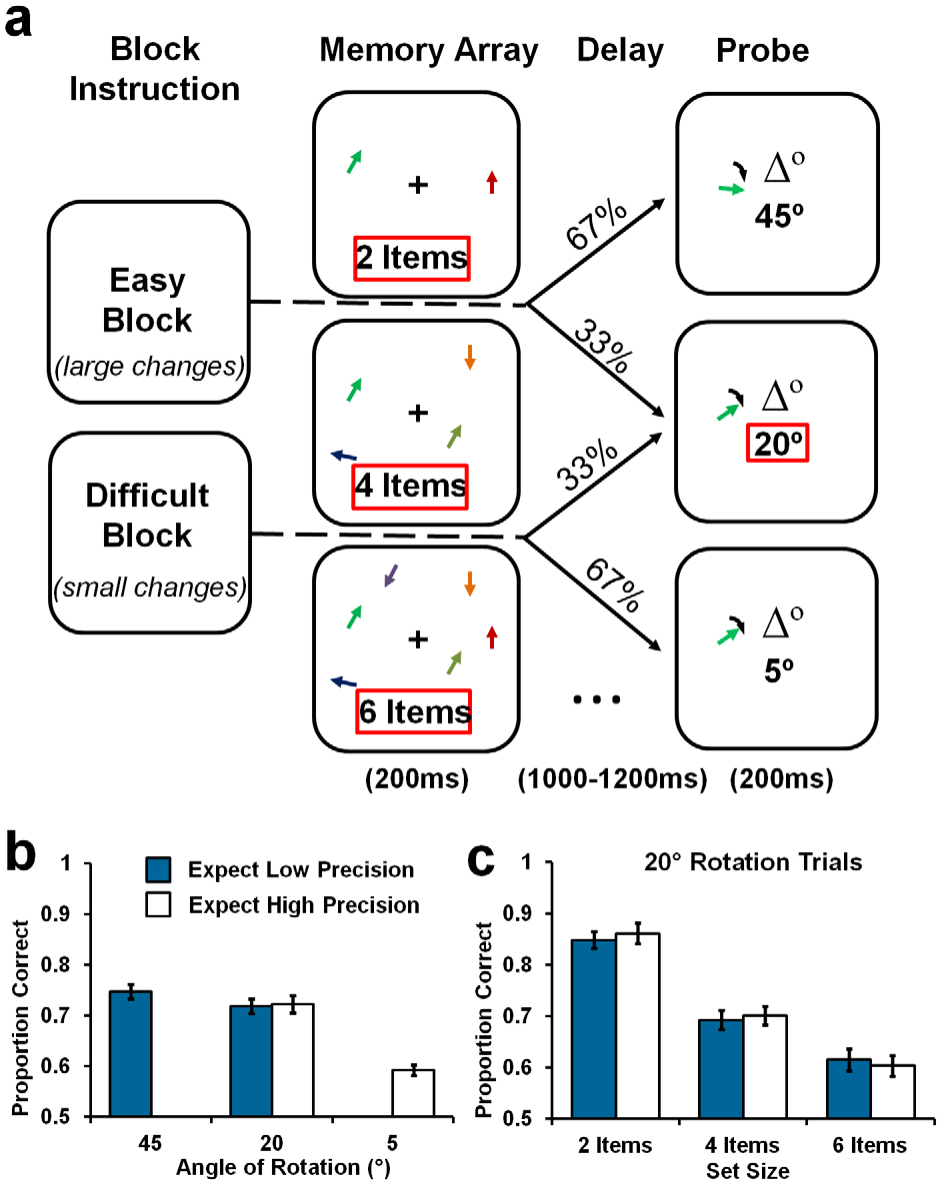
Condition schematic and main results for Experiment 3. [a] Schematic of the precision manipulation: for the *low precision* condition the Angular Change was probed at 20° or 45° (top). In the *high precision* condition, the Angular Change was probed at 5° or 20° (bottom). Red boxes indicate common comparison conditions between the levels of Block Type: Set Size (2, 4 and 6 items) and 20° Angle of Rotation. [b] The proportion of correct orientation change discriminations is shown as a function of Block Type and Angular Change. Data shown are the average across all trials. [c] The proportion of correct orientation change discriminations for the 20° Angle of Rotation condition as a function of Block Type and Set Size. Error bars represent ±1 *SEM*.

The conditions are illustrated in Figure 3a. The task design and stimulus parameters were the same as Experiment 2, with the exception that here we manipulated the expected degree of angular change, rather than the number of to-be-remembered items. For *expect low precision* blocks, the probe was rotated by 45° (66.67% of trials) or 20° (33.33% of trials). For *expect high precision* blocks, the probe rotated 20° (33.33% of trials) or 5° (66.67% of trials). This design enabled us to compare performance on a common condition (20° of Angular Change) to determine whether the prior knowledge of the likely precision required for the probe modulates the strategic allocation of VSTM resources. After collapsing over clockwise and anti-clockwise rotations, the resulting design manipulated three within-participant factors of Block Type (*expect low precision* vs. *expect high precision*), Angular Change (5° vs 20°, or 20° vs 45° depending on the block) and Set Size (2, 4 or 6 items). For full details of results see Supporting Information S3.

#### Procedure and analysis

Procedures, trial numbers and analyses were the same as Experiment 2, except participants were instructed that the cue information presented at the beginning of each block predicts the likely degree of angular change of the probe stimulus. The critical comparison was based on performance on the common level of Angular Change: 20°. Psychometric modelling was not possible for this experiment, because this requires a range of angular changes within the common condition.

### Results and Discussion

Like in the previous experiments, performance on the common 20° change condition did not differ as a function of Block Type [*F*_1,19_ = 0.14, *p = *0.72; see Figure 3b] and the interaction between Block Type and Set Size was not significant [*F*_2,38_ = 0.52, *p = *0.60]. There was a main effect of Set Size [*F*_2,38_ = 231.05, *p<*0.001], driven by significantly higher performance for the lower set sizes at every pairwise comparison (*p*s<0.001).

Again, there was no evidence that participants could use prior knowledge to influence the balance between the quality and the relative quantity of information in VSTM. Specifically, comparing accuracy at the common level of Angular Change (20°) across the two levels of Block Type suggested that being able to predict the optimal precision does not influence the way that resources are allocated.

In the experiments thus far, participants could predict the likely demands on quantity (Experiments 1 and 2) or quality (Experiment 3), but never both. In the next experiment, these characteristics were combined to maximise the possibility that participants could optimise the trade-off. In Experiment 4, if a high precision judgement is required, then only small set sizes will be presented. This should encourage participants to represent small arrays with high precision. Alternatively, if large arrays are presented then only coarse discriminations will be required. This should encourage participants to represent large arrays with low precision. The trade-off in task requirements should therefore provide a very strong test of the flexibility of the trade-off between the precision and number of representations.

## Experiment 4

Experiment 4 tested whether participants can optimally adjust the allocation of VSTM resources when the task fully encourages a trade-off between quality and quantity. Previously, the number and precision requirements were manipulated separately, which could have undermined the utility of the trade-off. The combination of the two characteristics maximises the potential advantage of preparatory allocation of memory resources for VSTM quality or quantity. *Quality* blocks encouraged encoding and maintenance of a low number of items with high precision, whereas *quantity* blocks emphasised maximising the number of representations in memory. In this experiment, the common condition was a combination of a particular number (four items) *and* precision (25°). If participants are able to flexibly fine-tune the quality/quantity trade-off, then performance on the common condition should be better in the *quality* condition.

### Methods

#### Participants

Twenty volunteers participated in Experiment 4 (14 female, 6 male; aged between 18 and 34). They had normal or corrected-to-normal vision and were right handed as assessed by a handedness inventory [21]. All participants gave informed written consent and were remunerated £10 for their participation.

#### Task and stimuli

**Figure 4:**
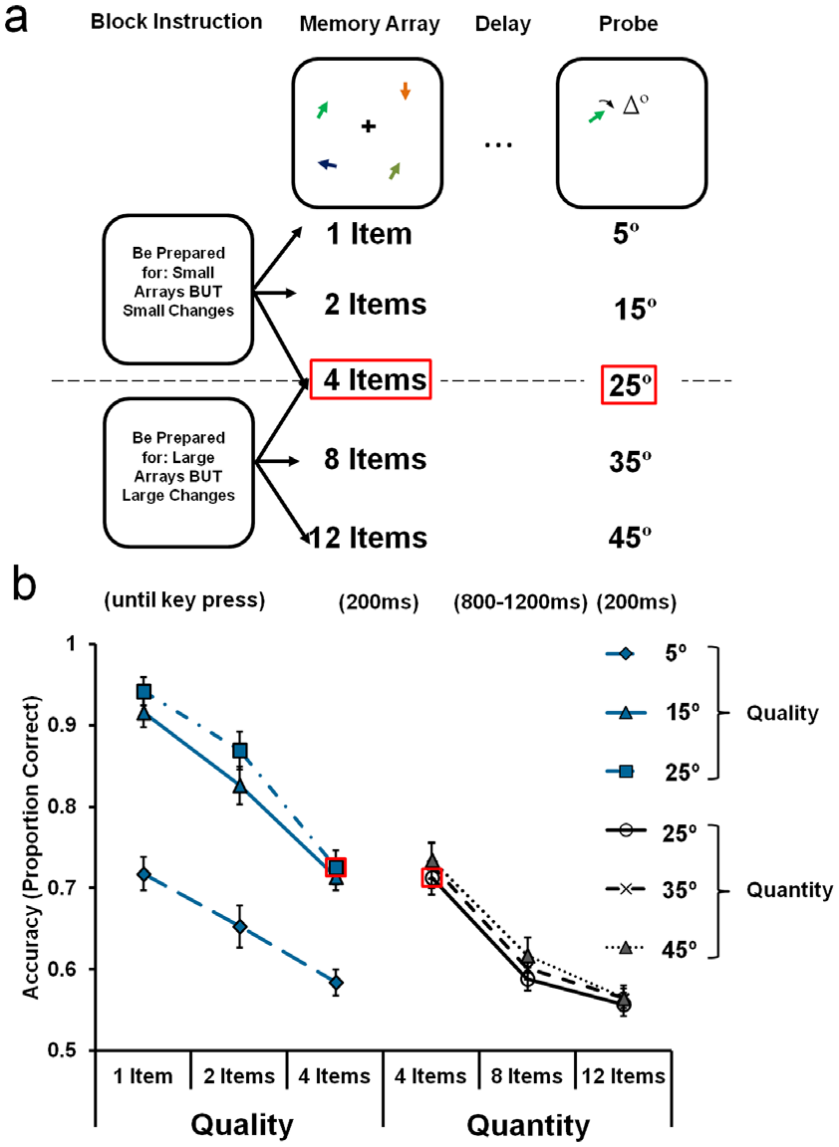
Condition schematic and main results for Experiment 4. [a] Schematic of the task manipulations. In the low load, high precision condition (*quality*) condition (top row), participants could expect 1, 2 or 4 items, and a probe rotated by either 5°, 15° or 25°. In the high load, low precision condition (*quantity*) condition, participants expected 4, 8 or 12 items, and a probe rotated by either 25°, 35° or 45°. [b] The proportion of correct orientation change discriminations is shown as a function of the Block Type. *Quality* is shown on the left, and *quantity* on the right. Data points represent accuracy as a function of Set Size (1, 2, vs. 4, or 4, 8 vs. 12 items) and Angular Change (5°, 15° vs 25°, or 25°, 35° vs 45°). Red boxes indicate common comparison conditions between the two conditions: 4 items and a 25° change. Error bars represent ±1 *SEM*.

The task design and stimulus parameters were similar to those in Experiments 2 and 3. The defining difference was that here we manipulated both the expected magnitude of angular change and the number of items in the array (see Figure 4a). Before each block, the text “Be Prepared for: Small Arrays BUT Small Changes” (Block Type: *quality*) or “Be Prepared for: Large Changes BUT Large Arrays” (Block Type: *quantity*) was presented in black, Arial 34pt font to cue participants about the likely precision and number of items in each trial for the upcoming block. In the *quality* blocks, there were one, two or four items in the array, and participants judged the direction of angular changes at 5°, 15° or 25°. In contrast, in the *quantity* blocks, the arrays contained 4, 8 or 12 items, and the probe rotated by 25°, 35° or 45° compared to the corresponding item in the memory array. Three levels of each Angular Change and Set Size were used to maximise the differences between the conditions, thereby maximising the potential utility of the contextual information. In total there were 1152 experimental trials.

As in all other experiments, this design enabled us to compare performance on a common condition (now with four items and at 25° rotation) to determine whether the prior knowledge of the combination of the number of items and the required precision can modulate the allocation of VSTM resources.

#### Procedure and analysis

Participants were instructed that the cue information presented at the beginning of each block predicts the likely number of items in the array *and* the degree of angular change of the probe stimulus. After collapsing over clockwise and anti-clockwise rotations, the resulting design manipulated three within-participant factors of Block Type (*quality* vs. *quantity*), Angular Change (5°, 15°, vs. 25°; or 25°, 35°, vs. 45° depending on the block) and Set Size (1, 2, vs. 4; or 4, 8, vs. 12 items depending on the block). The critical comparison was a paired samples t-test on performance for the common 4-item, 25° condition as a function of Block Type. For completeness, the effects of Angular Change and Set Size were analysed using a repeated measures *ANOVA* for the *quality* and *quantity* blocks separately (see Supporting Information S4).

### Results and Discussion

Critically, the comparison for the 4 item, 25° change condition (common across the two types of block), showed no significant effect of Block Type [*t*_19_ = 0.63, *p = *0.53; see Figure 4b]. Consequently, as in Experiments 1–3, there was no difference in VSTM performance between the two trade-off contexts. This null result, in combination with data from Experiments 1–3, suggests that participants did not trade off the quantity of items and the quality with which they are represented. Moreover, this final experiment shows that even directly encouraging the trade-off by setting the task context to require small arrays to be represented with high precision or large arrays to be represented with low precision could not affect performance. This eliminates the possibility that the null results in the previous experiments were due to the trade-off being undermined by encouraging one setting (e.g. high precision) without reducing the requirements to code the other (e.g. high set size). For example, in Experiments 1 and 2, it may not have been optimal to represent a large number of items with coarse precision because some trials still required fine discriminations. Likewise, in Experiment 3, some high precision trials still required participants to encode large numbers of items. In this experiment, however, such a conflict did not arise; participants were encouraged either to represent large arrays with low precision or small arrays with high precision.

## Discussion

Across four experiments, we found no evidence that participants use the provided strategic information to control the trade-off between the precision and number of items in VSTM. Specifically, we found no evidence that quality- or quantity-predictive information can influence the trade-off between these aspects of VSTM. These consistent null effects provide compelling evidence that top-down control mechanisms do not bias the trade-off of resource allocation for VSTM.

One possible reason to account for the lack of differences in the common condition across cue types in Experiment 1 was the inability to switch the strategic allocation of VSTM resources on a trial-by-trial basis. The additional cognitive demand of switching so frequently could have limited strategic top-down control in the allocation of VSTM resources. However, results from Experiments 2–4 suggest that limitations in the ability to change set size expectations rapidly, on a trial-by-trial basis were not sufficient to explain the null results in Experiment 1. We observed homologous results when expectations were blocked and when validity of manipulation increased.

The current study also provides convergent evidence that neither quantity nor quality predictions can be used to optimise the capacity trade-off in VSTM. Despite the similarity of the results and the task structure of Experiments 1–2, and Experiment 3, there is a fundamental asymmetry in manipulations of expected quantity and quality. When predictive information pertained to set size, the actual set size is immediately apparent at encoding and could override any prior expectations. If there is no advantage to preparing the mnemonic mechanisms in advance of the expected encoding demands, then resource allocation can simply wait until the memory array is presented. At that point, there is no difference between the contexts: VSTM resources can be deployed optimally for that trial. When the task expectations are defined by the required precision, however, the optimal trade-off cannot be ascertained from the memory array and is only apparent upon presentation of the probe. Consequently, expectation provides additional information that could optimise encoding, if the trade-off between quality and quantity were under flexible control. Surprisingly, this was not the case, especially in light of previous research demonstrating strong effects of top-down control of VSTM when a specific attribute (such as location) of a memory array should be prioritised e.g. [18].

Potentially, the results in Experiments 1–3 could be explained by an incomplete manipulation of quality/quantity cueing. In these experiments, the trade-off was encouraged in one setting (e.g. high precision) without reducing the requirements for the other (e.g. a high set size). For example, despite prior knowledge of a high set size, in some circumstances, participants were still required to make fine discriminations. As such, it may not have been advantageous to fine tune VSTM according to these predictions. We cannot, of course, rule out that some different and stronger changes in the task parameters and demands may have succeeded in shifting participants' strategies. However, we argue that the combination of the demand expectations in the final experiment created conditions of explicit and high motivation to set the trade-off between the number versus the precision of items to be encoded. In Experiment 4, either high resolution for a small array of items, or low-resolution representations of relatively large arrays was required. This combination should have provided a strong test for the flexible control over the trade-off between number and precision in VSTM. Consequently, we conclude that participants simply do not modulate the trade-off between quality and quantity in VSTM strategically, but rather, these resources seem to be allocated according to the stimulus-driven input.

The results of the current study are also complementary to the recently published study by Zhang and Luck [20]. These tasks were analogous to Experiment 3 in the current study because participants could predict how precisely they should encode the memory stimuli at encoding. Like the results presented here, Zhang and Luck [20] found that participants were unable to trade-off resolution and capacity in VSTM, even when given financial incentive to do so. In contrast, there was evidence of flexibility over the trade-off between quantity and quality of representations when memory was probed before the decay of the iconic trace. This is consistent with other important functional dissociations between VSTM and the high capacity, iconic trace e.g. [24]. Here we extend this previous research by manipulating the trade-off between the precision and *number* of representations (Experiment 4) and in doing so we provide important replications of our own and previously shown null-effects [20].

It seems likely that the flexibility in the control over the number and precision of mnemonic representations is lost during consolidation into the robust VSTM store [20], [25]. Zhang and Luck [20] suggest that the trade-off between the number and precision of items is only possible for information in a pre-VSTM representational form. Once items are encoded into robust VSTM, however, this flexibility is lost. Such an argument is akin to a *type/tokenised* account of processing, for a related discussion see [26]. Items in a pre-VSTM state are represented as *types,* which is relatively flexible. However, these representations need to be transformed, or *tokenised*, to form a stable item in VSTM. Once in VSTM, the number and precision of items is stable but no longer modifiable. The current study concurs with this idea, and shows that the biasing of VSTM resources in the trade-off between *quantity* and/or *quality* is not under flexible control. Zhang and Luck [20] show that expectation for precision can influence pre-VSTM representations. Future research could also explore whether set-size expectation also influences pre-VSTM.

The extent to which demand expectation could influence the trade-off between quality and quantity depends in part on the flexibility of the representational capacity of VSTM. A flexible resource model [1] makes a strong claim for a quality/quantity trade-off; however, a recent variant for a “fixed slot” model (e.g., fixed-slots + averaging model) also allows for some trade-off between number and precision of representations. In this account, at low set-sizes, items can occupy more than one slot, thereby improving representational quality [2]. At higher set sizes (>4 items), however, some theorists suggest that quality and quantity continue to trade-off [1], whereas others maintain that the number and precision of memory items remain fixed after the VSTM capacity is exceeded e.g. [2], [5].

In Murray et al. [18], we have previously highlighted the importance of modelling both slope and asymptote to estimate the number and precision of memory items at higher set-sizes when probed using the binary judgement change discrimination task. Memory quality and quantity at high set-size can also be estimated using a mixture model of angular error measured using a continuous judgement task [2]. However, if the continuous judgement response is made relative to a colour wheel as in [2], it is particularly important that participants accurately bind memory for location with the task-critical dimension (e.g., colour). Importantly, Bays, Catalao and Husain [8] demonstrate that binding errors in this task could artificially inflate the estimate of the guess rate, and correspondingly, the inferred number of items in VSTM. This is less problematic in our binary judgement, because participants can use space and/or colour of the stimulus to retrieve orientation information. Future research will help establish the key parameters that determine the trade-off between memory quality and quantity. Our data suggests that even if these two parameters interact, the trade-off is not influenced by demand expectation.

In summary, the current study finds no evidence for strategic top-down control over the trade-off between number and precision of representations to optimise the encoding and maintenance of behaviourally relevant information in VSTM. This result is in contrast with the high degree of flexibility to prioritise specific items during encoding [18]. Across four experiments, we find a resounding lack of evidence to suggest that participants strategically allocate limited VSTM capacity to favour coding many items coarsely, or few items precisely. Rather, it appears that the trade-off between the number and precision of items for encoding is determined with the physical presentation of the array of items to be encoded, overriding any prioritisation of one or other capacity-limiting factor.

## Supporting Information

Supporting Information S1 Supporting information for Experiment 1. *Table S1:* Performance (proportion correct) for expect low set-size and expect high set-size trials according to levels of Set Size and Angular Change.
(DOCX)

Supporting Information S2 **Supporting information for Experiment 2.** *Table S2:* Performance (proportion correct) for expect low set-size and high set-size blocks according to levels of Set Size and Angular Change.
(DOCX)

Supporting Information S3 **Supporting information for Experiment 3.** *Table S3:* Performance (proportion correct) for expect large change and expect small change blocks according to levels of Angular Change and Set Size.
(DOCX)

Supporting Information S4 **Supporting information for Experiment 4.**
(DOCX)
