# Supplementary material for: Lacking Control over the Trade-Off between Quality and Quantity in Visual Short-Term Memory
Source: PLoS One. 2012 Aug 8;7(8):e41223. doi: 10.1371/journal.pone.0041223 (PMC3414487; doi:10.1371/journal.pone.0041223)
Supplement: Supporting Information S1 — Supporting information for Experiment 1. Table S1: Performance (proportion correct) for expect low set-size and expect high set-size trials according to levels of Set Size and Angular Change. (DOCX) [file pone.0041223.s001.docx]

**Supporting Information S1**

Experiment 1

ANOVAs testing for effects of Set Size and Angular Change for trials of each Cue Type, revealed main effects of Set Size within each level of Cue Type. Accuracy was higher for 2-item arrays than 4-item arrays [*expect* *low* *set size*: *F*_1,11_ = 100.37, *p<*0.001; *expect high* *set size*: *F*_1,11_ = 5.25, *p=*0.043]. Main effects of Angular Change were also significant [*expect low set size*: [*F*_2,22_ = 68.10, *p<*0.001; *expect high* *set size*: *F*_2,22_ = 21.80, *p<*0.001]. Performance was significantly lower for 5° compared to 20° (*ps*<0.001), and 45° angles of rotation (*ps<*0.001), but was not different for 20° vs. 45° rotations (*ps>*0.29). Set size and Angular Change did not interact within either level of Cue Type (*p*s>0.20).

*Table S1:* Performance (proportion correct) for expect low set-size and expect high set-size trials according to levels of Set Size and Angular Change.

|  | Angular Change | | |
| --- | --- | --- | --- |
|  | 5°  (*SEM*) | 20°  (*SEM*) | 45°  (*SEM*) |
|  |  |  |  |
| **Expect Low Set Size Trials** |  |  |  |
| 2 Items | 0.69 (0.03) | 0.89 (0.01) | 0.91 (0.02) |
| 4 Items | 0.58 (0.02) | 0.71 (0.02) | 0.77 (0.02) |
|  |  |  |  |
| **Expect High Set Size Trials** |  |  |  |
| 4 Items | 0.61 (0.03) | 0.71 (0.02) | 0.74 (0.03) |
| 6 Items | 0.56 (0.03) | 0.67 (0.02) | 0.66 (0.01) |
