# Supplementary material for: Lacking Control over the Trade-Off between Quality and Quantity in Visual Short-Term Memory
Source: PLoS One. 2012 Aug 8;7(8):e41223. doi: 10.1371/journal.pone.0041223 (PMC3414487; doi:10.1371/journal.pone.0041223)
Supplement: Supporting Information S2 — Supporting information for Experiment 2. Table S2: Performance (proportion correct) for expect low set-size and high set-size blocks according to levels of Set Size and Angular Change. (DOCX) [file pone.0041223.s002.docx]

**Supporting Information S2**

Experiment 2

For completeness, performance was compared for each Set Size and Angular Change at each level of Cue Type. The proportion of trials across levels of Set Size conditions were not equivalent, therefore data were randomly sub-sampled to equate trial numbers.

Within each Cue Type, there were effects of Set Size [*expect* *low set-size:* *F*_1,19_ = 291.05, *p<*0.001; *expect* *high set-size: F*_1,19_ = 51.73, *p<*0.001] and Angular Change [*expect* *low set-size: F*_2,38_ = 146.02, *p<*0.001; *expect* *high set-size: F*_2,38_ = 24.70, *p<*0.001]. Performance was significantly higher for larger angles of rotation (*p*s<0.049). In each Cue Type, the interaction between Set Size and Angular Change was also significant [*expect* *low set-size*: *F*_2,38_ = 4.88, *p=*0.013; *expect* *low set-size*: *F*_2,38_ = 3.51, *p=*0.04]. For *expect* *low set-size* blocks, accuracy was significantly higher for 2 compared to 4 item trials for each level of Angular Change (*p*s≤0.001). For *expect* *high set-size* blocks, the differences in performance between the two Set Sizes were significant at both 45° (*p*<0.001) and 20° (*p*=0.006) but not at 5° (*p*>0.36).

*Table S2:* Performance (proportion correct) for expect low set-size and high set-size blocks according to levels of Set Size and Angular Change.

|  | Angular Change | | |
| --- | --- | --- | --- |
|  | 5°  (*SEM*) | 20°  (*SEM*) | 45°  (*SEM*) |
|  |  |  |  |
| **Expect Low Set Size Blocks** |  |  |  |
| 2 Items | 0.64 (0.02) | 0.86 (0.02) | 0.91 (0.02) |
| 4 Items | 0.55 (0.02) | 0.70 (0.02) | 0.74 (0.02) |
|  |  |  |  |
| **Expect High Set Size Blocks** |  |  |  |
| 4 Items | 0.57 (0.01) | 0.68 (0.03) | 0.73 (0.02) |
| 6 Items | 0.55 (0.02) | 0.61 (0.02) | 0.63 (0.02) |
