# Supplementary material for: Lacking Control over the Trade-Off between Quality and Quantity in Visual Short-Term Memory
Source: PLoS One. 2012 Aug 8;7(8):e41223. doi: 10.1371/journal.pone.0041223 (PMC3414487; doi:10.1371/journal.pone.0041223)
Supplement: Supporting Information S3 — Supporting information for Experiment 3. Table S3: Performance (proportion correct) for expect large change and expect small change blocks according to levels of Angular Change and Set Size. (DOCX) [file pone.0041223.s003.docx]

**Supporting Information S3**

Experiment 3

In both levels of Block Type, there were main effects of Angular Change [*expect* *large change*: *F*_1,19_ = 5.01, *p=*0.037; *expect* *small change*: *F*_1,19_ = 120.43, *p<*0.001] and Set Size [*expect* *large change*: *F*_2,38_ = 169.55, *p<*0.001; *expect* *small change*: *F*_2,38_ = 104.69, *p<*0.001]. For both levels of Block Type, lower set sizes were associated with higher performance at every comparison (*p*s<0.001). The interaction between Angular Change and Set Size was also significant in both cases [*expect* *large change*: *F*_2,38_ = 3.46, *p=*0.042; *expect* *small change*: *F*_2,38_ = 21.71, *p<*0.001]. For *expect* *large change* blocks, the difference in accuracy between the two levels of Angular Change was only significant for the 2-item trials [*p*<0.001; other comparisons: *ps*>0.17]. In contrast, the differences in accuracy between levels of Angular Change were significant for each set size (*p*s≤0.02) for the *expect* *small change* blocks.

*Table S3:* Performance (proportion correct) for expect large change and expect small change blocks according to levels of Angular Change and Set Size.

|  | Set Size | | |
| --- | --- | --- | --- |
|  | 2 Items (*SEM*) | 4 Items  (*SEM*) | 6 Items (*SEM*) |
|  |  |  |  |
| **Expect Large Change Blocks** |  |  |  |
| 45° | 0.90 (0.01) | 0.72 (0.02) | 0.61 (0.02) |
| 20° | 0.85 (0.02) | 0.69 (0.02) | 0.62 (0.02) |
|  |  |  |  |
| **Expect Small Change Blocks** |  |  |  |
| 20° | 0.86 (0.02) | 0.70 (0.02) | 0.60 (0.02) |
| 5° | 0.65 (0.02) | 0.58 (0.02) | 0.55 (0.02) |
