# Supplementary material for: Lacking Control over the Trade-Off between Quality and Quantity in Visual Short-Term Memory
Source: PLoS One. 2012 Aug 8;7(8):e41223. doi: 10.1371/journal.pone.0041223 (PMC3414487; doi:10.1371/journal.pone.0041223)
Supplement: Supporting Information S4 — Supporting information for Experiment 4. (DOCX) [file pone.0041223.s004.docx]

**Supporting Information S4**

Experiment 4

Analysis of accuracy at both levels of Block Type revealed the expected decrease in performance for higher Set Sizes [*quality*: *F*_2,38_ = 112.27, *p<*0.001; *quantity*: *F*_2,38_ = 69.06, *p<*0.001; for all pairwise comparisons, *p*s≤0.001]. Angular Change was only significant for the *quality* block [*quality*: *F*_2,38_ = 125.63, *p<*0.001; *quantity*: *F*_2,38_ = 1.54, *p=*0.23]. In the *quality* block, there was significantly higher performance for larger angles at every comparison (*p*s<0.03). The interaction between Set Size and Angular Change was also only significant for the *quality* block [*quality*: *F*_4,76_ = 3.93, *p=*0.006; *quantity*: *F*_4,76_ = 0.20, *p=*0.94]. In the *quality* block, the differences in accuracy between levels of Set Sizes were significant for all levels of Angular Change [low set size>high set size; all *p*s<0.024].
